# Teratoma of the anterior mediastinum presenting as a cystic neck mass: a case report

**DOI:** 10.1186/1752-1947-2-23

**Published:** 2008-01-28

**Authors:** Gaurav Agarwal, Dilip K Kar

**Affiliations:** 1 Department of Endocrine Surgery, Sanjay Gandhi Postgraduate Institute of Medical Sciences, Raebareli Road, Lucknow- 226014, India; 2 Dept of Surgical Oncology, JLN Cancer Hospital, Bhopal, India

## Abstract

**Introduction:**

Teratomas of anterior mediastinum are rare tumors and are often slow growing, asymptomatic and detected incidentally on chest imaging. Results of surgical resection are very satisfactory.

**Case presentation:**

A 19-years old male presented with an asymptomatic cystic neck mass. X-ray and CT scan of chest and neck showed an extrathyroidal multi-septate, predominantly cystic neck mass, that was continuous with a solid intrathoracic mass extending up to the level of right atrium and which contained areas of calcification and cystic necrosis. The mediastinal structures did not show any features of compression or infiltration. Fine needle aspiration cytology from the neck mass was suggestive of a dermoid cyst. In view of the extent and uncertain pathological nature of the tumor, it was excised via a combined cervical and trans-sternal route. Histo-pathology of the resected specimen confirmed the diagnosis of a mature cystic teratoma. The patient made an uneventful recovery, and after five years of follow-up, he has been symptom free with no clinical or radiological evidence of recurrent disease. We discuss the role of imaging and the need for surgical treatment to avoid possible catastrophic complications in patients with cervical and mediastinal masses of uncertain histological nature.

**Conclusion:**

A mediastinal teratoma may rarely present as a cystic neck swelling due to its cephalad extension. This entity needs to be considered in cases where clinical and investigative work-up fail to provide a convincing clue to a primary neck pathology as cause of a cystic neck swelling.

## Introduction

Teratomas of the anterior mediastinum account for 8–13% of tumors in this region [1]. The majority of these teratomas are located in the anterior mediastinum with only 3–8% arising from the posterior mediastinum [2-4]. These slow growing tumors are often asymptomatic and are often detected incidentally on chest radiographs. Complications such as atelectasis, adhesion to, or compression of, adjacent structures, or malignant transformation are occasionally encountered. Results of surgical resection are usually very satisfactory.

We report the case of a young adult male who presented with a cystic neck mass, due to degeneration of a cervical extension of a mature teratoma of the anterior mediastinum. This is an unusual presentation of an uncommon pathology.

## Case presentation

A 19 years old male presented with a rapidly progressing painless neck swelling of 3 months duration. There were no complaints of fever or weight loss or symptoms suggestive of compression of adjacent structures. The patient was afebrile and had a cystic, non-tender, non-translucent and smooth swelling occupying the whole of the neck anteriorly, extending from the hyoid bone to the suprasternal notch. The lower limit of the swelling could not be reached. There were no neck nodes or dilated tortuous neck or chest wall veins.

**Figure 1:**
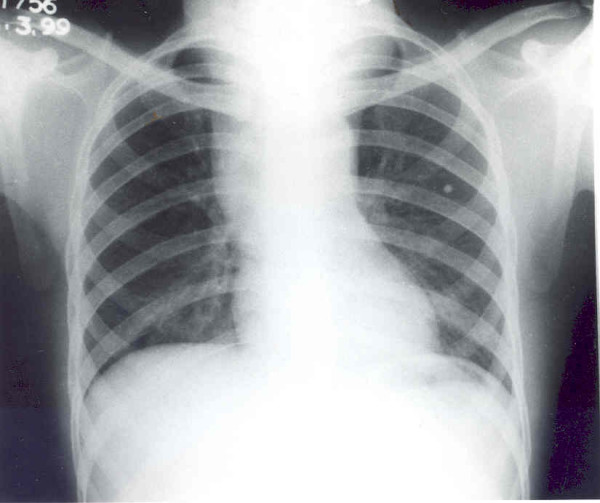
Chest x-ray (P-A view) showing widening of the mediastinum and a soft tissue swelling in the neck, continuous with the mediastinal shadow.

**Figure 2:**
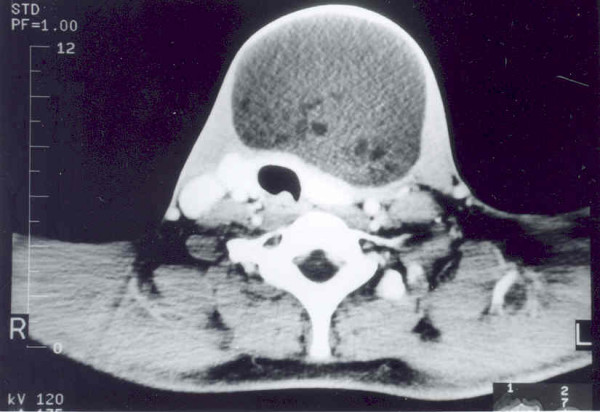
Contrast-enhanced CT scan of the neck showing a multi-septate cystic neck mass, lying superficial to the thyroid lobes and strap muscles.

**Figure 3:**
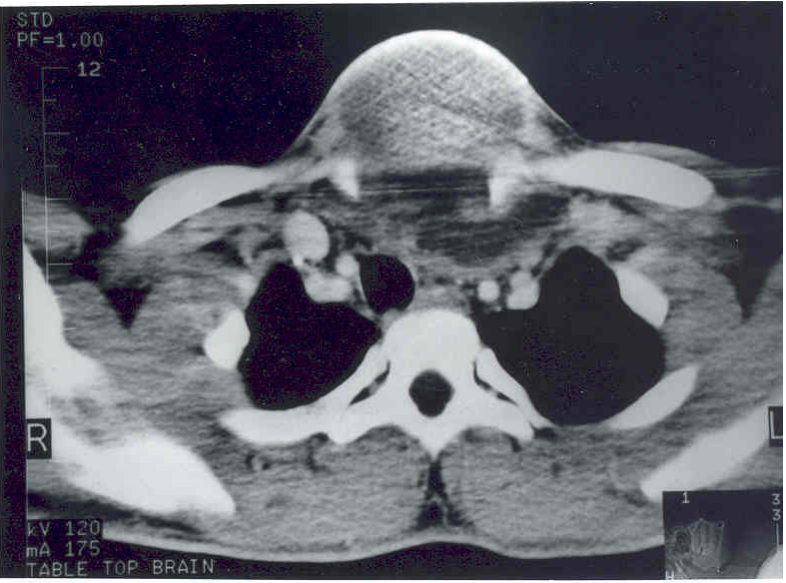
Contrast-enhanced CT scan of the upper thorax showing extension of a complex cystic mass and displacement of the trachea with compression of major vessels.

Chest X-ray revealed widening of the mediastinum and a soft tissue neck swelling, continuous with the mediastinal shadow (Fig 1). On neck ultrasonography, a predominantly cystic, multiseptate mass lying superficial to the thyroid lobes was evident. Fine needle aspirate cytology from the swelling showed largely necrotic material with few macrophages and mixed inflammatory cells on a background of proteinacious material, suggesting a diagnosis of a dermoid cyst. There were no acid-fast bacilli, bacterial and fungal elements on microscopy and culture. ^99m^Tc-thyroid scan ruled out a thyroid lesion. Contrast enhanced CT of neck and mediastinum (Fig 2 and 3) showed a multi-septate, predominantly cystic neck mass, superficial to thyroid and strap muscles. The mass was continuous with a more solid intrathoracic mass with areas of calcification and cystic necrosis, extending up to the level of right atrium. The trachea was shifted to the right but there was no compression or infiltration of great vessels or mediastinal structures. There were no mediastinal lymph nodes and pulmonary lesions. A diagnosis of anterior mediastinal teratoma with cervical extension was made.

The patient was operated upon through a neck crease and median sternotomy incision under general anesthesia with endotracheal intubation. The posterior wall of the mass in the neck was adherent to the strap muscles. A solid mediastinal tumour with areas of necrosis, which seemed to be arising from the thymus gland, was found on sternotomy. The tumor derived its blood supply from the thoracic vascular channels as direct branches from aortic arch and the subclavian artery. There were no vessels feeding or draining the tumor in the neck. The mass was adherent to but did not infiltrate, the innominate vein. The neck and thoracic mass, along with a densely adherent 4 × 3 cm area of left mediastinal pleura, were removed in continuity, preserving nearby vital structures.

The resected surgical specimen was subjected to a detailed histopathological evaluation. On naked eye examination, the globular brownish mass measured 16 × 7 × 2 cms. The cut surface of the mass was cystic, filled with yellow pultaceous material and a mass of hair. There was a solid area projecting into the lumen of the cystic area, which had multiple cysts filled with gelatinous material. On microscopy, the cyst wall showed predominantly degenerate necrotic area, associated with inflammatory cells. Sections from the solid areas revealed cartilage, osseous tissue and nerve bundles with ganglionic cells, respiratory epithelium and sero-mucinous glands embedded in dense fibro-collagenous and fibro-muscular tissue. The lesion was thus labeled as a mature cystic teratoma on basis of the histopathological features.

The patient made an uneventful postoperative recovery. After five years in the follow-up, the patient has remained asymptomatic, and follow-up imaging studies in the form of chest x-ray examination undertaken 3, 14 and 36 months after operation, as well as a contrast enhanced CT scan of the neck and mediastinum performed 14 months after operation, have not shown any residual or recurrent mass.

## Discussion

Teratomas are congenital tumors that contain derivatives of all three germ layers and arise from pluripotent embryonal cells. They commonly occur in ovaries, testes, retroperitoneum and the sacro-coccygeal region. Superior mediastinal teratomas are usually asymptomatic till late, and are often discovered incidentally on chest x-ray. Symptoms such as chest pain, dyspnoea or cough are a result of compression of nearby structures. Rarely, the teratoma may rupture into tracheo-bronchial tree or result in SVC syndrome or pneumonia [5]. The interesting feature of our patient was that he presented with a rapidly enlarging, yet other wise asymptomatic, neck mass. Perhaps it was because the mediastinal mass found an escape route into the neck, tha our patient did not have features of mediastinal compression, despite the large tumor size.

Chest x-ray is an important aid in diagnosis of a mediastinal teratoma. Mediastinal CT scan demonstrates the extent of a mass better than conventional radiography. It can also detect fatty or cystic areas in mediastinal masses, but this information will not obviate the need for surgical resection to establish the final diagnosis [4]. CT scan is helpful in defining invasion of adjacent structures and thus assists planning surgical intervention [5]. CT scan (Fig 2 and 3) of neck and mediastinum in our patient established the continuity of mediastinal mass into the neck and detected adherence of the mass to pericardium.

Complete curative surgical removal of a mediastinal teratoma is the treatment of choice, as it establishes the diagnosis, besides preventing life threatening complications in many patients [6]. Malignant mediastinal teratomas account for roughly 1–5% of all mediastinal tumors [7,8]. Invasion or great vessels, myocardium, lung and phrenic nerves should be taken as indicators of malignancy, and may necessitate extensive operation in selected patients [7]. Complications of extensive surgical procedures such as pneumonectomy, rather than the disease itself, may prove fatal [4]. Adherent mediastinal pleura and pericardium can be dealt with by removal of the involved portions. As most mediastinal teratomas are benign, even a subtotal resection conserving adherent vital structures provides excellent results. In present era of modern surgical practices, excellent outcome has been the rule [5].

Our patient represents an unusual presentation of this not so uncommon pathological entity. Extension of a mediastinal teratoma into the neck and its cystic degeneration gave rise to this presentation. A search of the English language medical literature failed to find many similar cases.

## Conclusion

A cystic neck swelling may rarely be caused by cephalad extension of a mediastinal teratoma. This entity needs to be considered in cases where clinical and investigative work-up fail to provide a convincing clue to a primary neck pathology as cause of a cystic neck swelling.

## Abbreviations

CT: Computed tomography

FNAC: Fine needle aspiration cytology

## Competing interests

The author(s) declare that they have no competing interests.

## Authors' contributions

GA conceived the case report, contributed to collection of clinical details and writing, reviewing and finalization of the manuscript; DKK prepared the first draft besides contribution to collection of clinical details and illustrations. Both authors reviewed and finally approved the final manuscript.

## Consent

Written informed consent was obtained from the patient for publication of this case report and accompanying images. A copy of the written consent is available for review by the Editor-in-Chief of this journal.
